# A need-based, multi-level, cross-sectoral framework to explain variations in satisfaction of care needs among people living with dementia

**DOI:** 10.1186/s12913-020-05416-x

**Published:** 2020-07-15

**Authors:** Chiara De Poli, Jan Oyebode, Mara Airoldi, Richard Glover

**Affiliations:** 1 Department of Social Policy and Department of Management, London School of Economics and Political Science, Houghton Street, London, WC2A 2A UK; 2 Centre for Applied Dementia Studies, Faculty of Health Studies, Richmond Road, Bradford, BD7 1DP UK; 3 Blavatnik School of Government, University of Oxford, Radcliffe Observatory Quarter, 120 Walton St, Oxford, OX2 6GG UK; 4 NHS North of England Commissioning Support, John Snow House, Durham, DH1 3YG UK

## Abstract

**Background:**

Provision of care and support for people with dementia and family carers is complex, given variation in how dementia manifests, progresses and affects people, co-morbidities associated with ageing, as well as individual preferences, needs, and circumstances. The traditional service-led approach, where individual needs are assessed against current service provision, has been recognised as unfit to meet such complexity. As a result, people with dementia and family members often fail to receive adequate support, with needs remaining unmet. Current research lacks a conceptual framework for explaining variation in satisfaction of care needs. This work develops a conceptual framework mapped onto the care delivery process to explain variations in whether, when and why care needs of people with dementia are met and to expose individual-, service-, system-level factors that enable or hinder needs satisfaction.

**Methods:**

Data collected through 24 in-depth interviews and two focus groups (10 participants) with people with dementia and family carers living in the North East of England (UK) were analysed thematically to develop a typology of care needs. The need most frequently reported for people with dementia (i.e. for support to go out and about) was analysed using themes stemming from the conceptual framework which combined candidacy and discrepancy theories.

**Results:**

The operationalisation of the framework showed that satisfaction of the need to go out was first determined at the point of service access, affected by issues about navigation, adjudication, permeability, users’ resistance to offers, users’ appearance, and systems-level operating conditions, and, subsequently, at the point of service use, when factors related to service structure and care process determined (dis)satisfaction with service and, hence, further contributed to met or unmet need.

**Conclusion:**

The conceptual framework pinpoints causes of variations in satisfaction of care needs which can be addressed when designing interventions and service improvements.

## Background

Provision of care for people living with dementia is complex, given considerable variation in how dementia manifests, progresses and affects people, often alongside co-morbidities associated with ageing, and how personal preferences and family circumstances vary. Such complexity determines the diversity of care needs that people experience, ranging from more basic (e.g. help with self-care) [1] to practical (e.g. help with managing own finances) and psycho-social needs (e.g. being accepted as part of a community) [2, 3]. Moreover, it determines the configuration of care provision, which requires inputs from different sectors (e.g. health, social care, third sector), different organisations within the same sector (e.g. mental and acute health care) and family and friends (‘informal’ carers) who play a major role in supporting people with dementia and reduce the pressure (also financial) on formal services [4–6].

Unsurprisingly given this complexity, actual provision of dementia care is known for being suboptimal and leaving individuals’ needs unmet [7–13]. Reasons for this are manifold. Missed or delayed diagnosis may leave those who are not diagnosed without support and treatment [14]. Lack of information may prevent people from accessing local services [7, 12, 13]. Post-diagnostic support and care, when available, may be fragmented and poorly coordinated leading to service duplications or gaps, difficult transitions between services (e.g. from hospital to community care) [15] and ineffective ways of linking formal and informal care [5], leaving people falling through the cracks of the system. Reluctance to seek assistance from services which are perceived to be poorly aligned with individual’s cultural values [16–18] or to be inconvenient for very practical reasons (e.g. because of costs or opening hours) [7, 13, 17–20] may further hinder service access. These factors may explain why people with dementia report different degrees of satisfaction for similar types of services and why needs may remain unmet.

In this article we argue that a systematic understanding of the reasons for unmet needs among people with dementia is currently lacking and we present a conceptual framework to explain the variation in whether, when and why needs are met. The framework incorporates the candidacy theory of access [21] and the discrepancy theory of patient satisfaction [22, 23], mapped on to the service delivery system. In doing so, it identifies points in the system where variation arises and pinpoint individual-, service- and system-level factors which can explain this variation. Moreover, it adopts a need-based approach to dementia care, pivoted around individual’s needs *per se* and irrespective of the services currently available [24], hence overcoming the traditional service-led approach, where individual needs are assessed with respect to current service provision and defined eligibility criteria. Being a need-based framework, its operationalisation necessarily spans across care organisations and sectors and is not constrained within organisational boundaries.

The article firstly presents the theoretical roots and key components of the conceptual framework and, then, discusses how it has been operationalised with respect to a need commonly reported by people with dementia, i.e. the need for support to go out and about. The results show the potential of the framework to provide professionals and policy-makers with insights around enablers and barriers to the provision of dementia care that lives up to the person-centredness discourse embedded in the current global [25] and English dementia improvement agenda [26, 27]. Equally, the conceptual framework makes a theoretical contribution to the field of evaluation and implementation research. By offering a comprehensive configuration of the factors that determine the satisfaction of care needs, the framework constitutes a theory that can inform the design, implementation and evaluation of interventions.

### Conceptual framework

The conceptual framework is built around two analytical components. The first one is represented by the care delivery process, which is unpacked in five consecutive phases

1. Identification of a need
2. Availability of a service which could satisfy the identified need
3. Access to the identified service
4. Utilisation of the accessed service
5. Satisfaction of the need identified

**Fig. 1:**
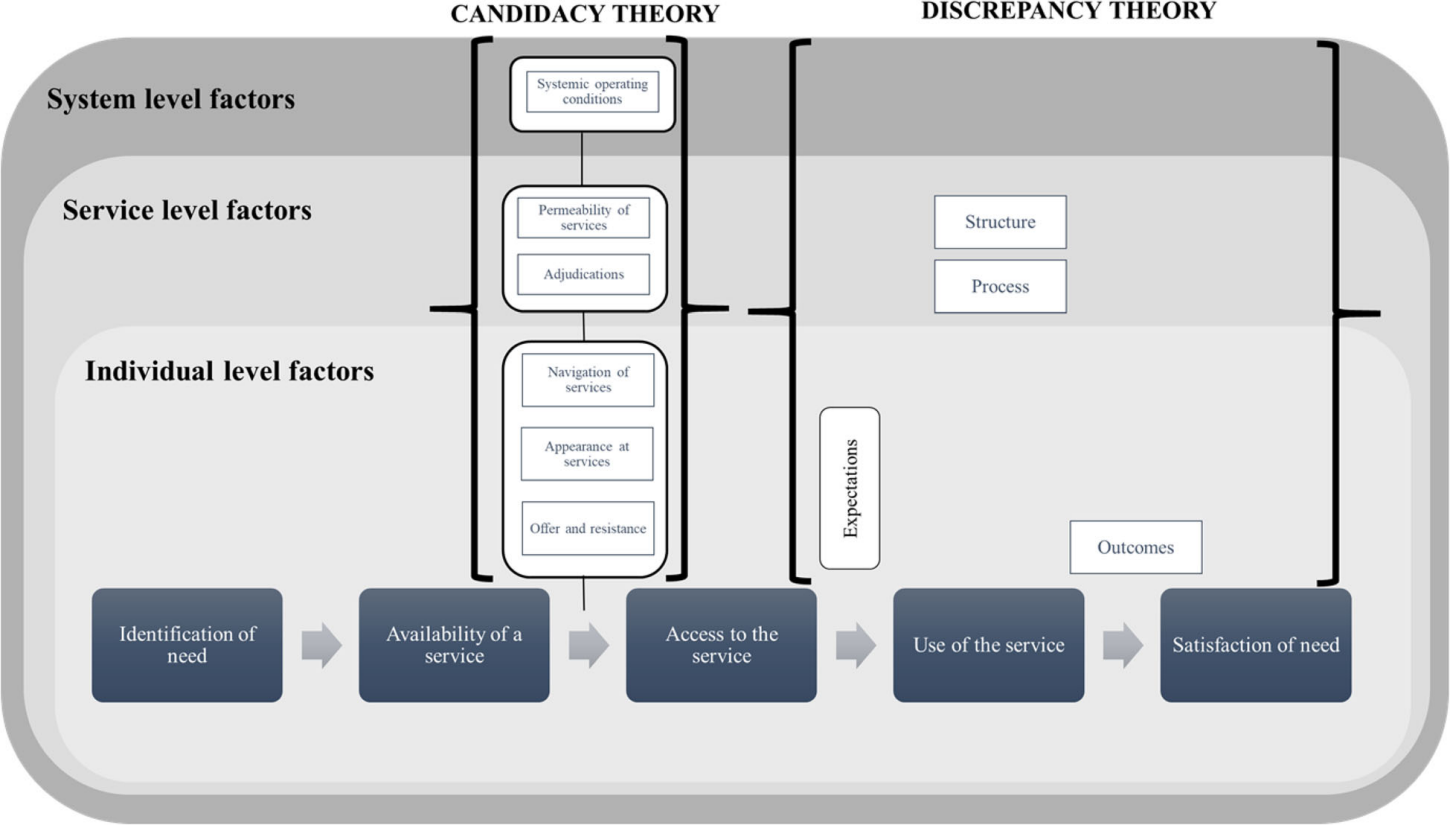
The conceptual framework

The second component is represented by the set of factors that influence the process of seeking, accessing and using care and operates at the individual (e.g. individual and family preferences and circumstances), service (e.g. delivery mode, referral criteria) and system levels (e.g. resource allocation, national regulation, local policies) (Fig. 1).

The complexity of the framework is amplified by the type of population to which it is applied. For example, populations with multiple, complex needs may require care from several services from different sectors. When the care process is poorly coordinated, the process of service delivery is repeated across the set of organisations involved. Moreover, individuals with progressive conditions may have to renegotiate care access and provision from different configurations of services as their needs change over time.

Conceptually, the framework integrates candidacy theory [21] and discrepancy theory [22, 23] and aims to highlight the gap between service availability and service access (as per candidacy theory) and between service use and user satisfaction (as per discrepancy theory).

“Candidacy describes the ways in which people’s eligibility for medical attention and intervention is jointly negotiated between individuals and services” [22: 41]. The amount, difficulty, and complexity of service negotiation may operate as a barrier to care access, with access being the outcome of this negotiation process, if successful.

The concept of candidacy challenges traditional utilisation approaches to care which conflate access to care with utilisation of care, postulating that availability of services will lead to their utilisation, with access uncritically assumed to be possible [28]. This view fails to acknowledge that access is influenced not only by supply (availability of a service) but also by demand (whether a service is sought) [29]. It overlooks that access requires a ‘degree of fit’ between services and service users [30], conditional on providers knowing what service would be acceptable to users, and on service users’ (or close others’) recognition of their own needs, influenced by their perceptions of illness and previous health care experiences. Finally, it focuses on system entry rather than on the outcome of such entry: access in itself is not sufficient evidence of needs being met [28].

Candidacy theory unpacks the concept of access by taking into account its dynamic and contingent character [21]. Candidacy may be affected over time through one’s social and cultural circumstances, personal experiences of service use and repeated encounters with professionals [29]. It is defined and redefined through situated interactions between individuals and professionals and can be determined by locally specific factors [21]. Hence, candidacy is shaped in an ongoing process of negotiation, continuously influenced by intervening events rather than being definitively determined in the context of a specific event or at a single point of service access [29].

Candidacy theory highlights the multi-dimensional nature of access, which is seen as subject to seven conditions which we have placed at individual-, service- and system-levels (Fig. 1). These influence how one individual candidacy (i.e. the negotiated individual eligibility to access) is framed and managed. The first six can be viewed as transition points at which a person’s candidacy for care must be negotiated, and the seventh captures the broader context in which negotiations unfold [31].

*Identification* of candidacy refers to recognition that a need requires intervention. In our framework this condition is seen as a prerequisite to service delivery and is considered as a separate first stage. *Navigation* highlights the ease with which service access can be navigated by potential users, considering the cognitive (e.g. being aware of the services on offer) and practical (e.g. being able to mobilise the resources – transport, finances, time –required to access services) efforts required of them. *Appearance* at services pinpoints how, in order to access a service, a potential user is expected to deploy a set of competencies to articulate credibly their needs or reasons for seeking help. Being unable to do so may hinder or limit access. *Offer and resistance* refers to the response of a potential user to the offer of accessing a service. Non-utilisation may be a consequence of a non-offer or the deliberate choice of resisting that offer. *Permeability* of services refers to the degree of alignment between users and services, for example determined by the eligibility criteria for referral into a service or by pragmatic considerations, such as service hours of operation or language. *Adjudications* refer to the judgements and decisions made by professionals with respect to a candidacy claim. Adjudications unfold at the service-level, but can be affected by the operating conditions in which professionals work (including resource constraints), by the dominant public discourse around entitlement of specific population groups (e.g. migrants), and by professional’s subjective perceptions about the appropriateness of the intervention for the possible user and consideration of her social deservingness [21]. Lastly, favourable *system-level conditions* determined by the availability and suitability of local resources are seen as crucial for the production of candidacy [21].

In our framework, candidacy theory is supplemented by discrepancy theory, an expectation-based approach to the evaluation of patient satisfaction. In traditional patient satisfaction theory, satisfaction is related to the perception of the benefits of care and the extent to which care meets individual expectations. In the simplest form, (dis)satisfaction is viewed as a reflection of the difference between what is expected and what is perceived to have been delivered [32]. Discrepancy theory defines satisfaction as the difference between individual expectations and actual experience, as a proportion of individual expectations [22]. Subsequent theoretical refinements stemming from Donabedian’s quality of care framework allow definition of the content of expectations in terms of structure (e.g. facilities, personnel), process (e.g. professionals’ competency and their communication skills), and outcomes (somatic and psychological), to reflect the efficacy of the service and the extent to which it was perceived to benefit its users [33].

The conceptual framework uniquely integrates two theories which operate consistently at three different levels (i.e. individual, service, system) while focussing on subsequent points of the care delivery process. In doing so, the framework mitigates some of the limitations intrinsic to the theories when used in isolation. Each theory not only makes some unsatisfactory simplifying assumptions (e.g. candidacy theory assumes that a need is recognised, however this may not always be the case), but also focuses on one phase of the care delivery process (e.g. candidacy theory focusses on access) and, consequently, can only provide explanations for unmet needs arising in that specific phase. The integrative approach of the framework overcomes these tensions and allows expansion of the explanatory power of the two individual theories.

## Methods

Following ethical and research governance approvals from the West Midlands - South Birmingham Research Ethics Committee [REC reference 16/WM/0397], data were collected through 24 in-depth, semi-structured interviews and two focus groups with people living with dementia and family carers in the North East of England (UK). The interview and focus group guides are provided as Additional files 1, 2 and 3.

Interviews were conducted with a purposeful sample recruited during 2017 through general practitioners, third-sector organisations, local commissioners and care homes. Participants were invited to take part if they had received a diagnosis of dementia or cared for somebody living with dementia. The Mental Capacity Act 2005 was applied when recruiting participants with dementia and throughout the research process and only those with capacity to consent were invited to join the study.

We aimed for maximum variation in living arrangements, place of residence and socio-economic status. We included two family carers whose relative had died about 12 weeks before the interview, as their experience of dementia care was recent. Interviews lasted on average an hour. Seventeen were with family carers, three with a person living with dementia and four with both the family carer and the person living with dementia when the latter needed support to take part (Table 1).

**Table 1:** Characteristics of interviewees

		Family carer			Person with dementia (PWD)					
Id	Interviewee	Age	Gender	Relation with PWD	Age	Gender	Years from diagnosis	Type of diagnosis	Disease severity	Geography
CDP01	Carer	68	Female	Daughter	93	Female	1	Mixed	Moderate/severe	Urban
CDP02^a^	Carer	54	Female	Daughter	86	Male	4	Alzheimer’s	Moderate/severe	Urban
CDP03	Carer	56	Male	Son	77	Female	5	Alzheimer’s	Moderate/severe	Urban
CDP04	Carer	69	Female	Partner	74	Male	2	Lewy body	Moderate	Urban
CDP05	Carer	69	Female	Spouse	76	Male	9	Alzheimer’s	Moderate	Urban
CDP06	Carer	57	Female	Daughter	81	Female	4	Alzheimer’s	Moderate	Rural
CDP07	Couple				80	Female	4	Alzheimer’s		Rural
CDP08	PWD				72	Female	4	Mixed dementia		Rural
CDP09	Carer	53	Female	Daughter-in-law	79	Female	5	Mixed	Mild/moderate	Urban
CDP10	PWD				82	Male	1	Not known		Rural
CDP11	Carer	74	Female	Spouse	80	Male	8	Mixed	Severe	Rural
CDP12	Carer	68	Female	Spouse	77	Male	22	Vascular	Severe	Rural
CDP13	PWD				79	Male	1	Not known		Rural
CDP14	Carer	71	Female	Spouse	73	Male	9	Vascular	Severe	Rural
CDP15	Couple				80	Female	1	Vascular		Rural
CDP16	Carer	59	Female	Spouse	66	Male	3	Lewy body	Mild/moderate	Urban
CDP17^a^	Carer	70	Female	Spouse	72	Male	NA	Vascular	Severe	Rural
JO01	Carer	87	Male	Spouse	85	Female	6	Not known	Moderate	Urban
JO02	Carer	69	Male	Spouse	65	Female	2	Alzheimer’s	Moderate	Rural
JO03	Carer	68	Female	Spouse	81	Male	9	Mixed	Moderate	Rural
JO05	Couple				71	Female	2	Vascular		Rural
JO06	Carer	57	Female	Daughter	86	Male	2	Mixed	Severe	Urban
JO07	Carer	63	Female	Daughter	85	Female	2	Vascular	Moderate	Rural
RG01	Couple				86	Male	3	Vascular		Rural

^a^deceased

For the focus groups, organised through local third-sector organisations, similar inclusion criteria were used. In total, seven family carers and three people living with dementia attended (Table 2). The focus groups lasted about 90 min. The first took place in a community space (November 2017), the second on the premises of the hosting organisation (February 2018).

**Table 2:** Characteristics of focus groups participants

		Family carer			Person with dementia (PWD)					
ID	Interviewee	Age	Gender	Relation with PWD	Age	Gender	Years from diagnosis	Type of diagnosis	Disease severity	Geography
FG01	Carer	64	Female	Spouse	66	Male	<1 year	Alzheimer’s	NA	Rural
FG02	PWD				66	Male	<1 year	Alzheimer’s	Mild	Rural
FG03	Carer	40	Female	Daughter	80	Female	2	Alzheimer’s	Mild/moderate	Rural
FG04	Carer	55	Male	Son	83	Female	<1 year	Alzheimer’s	Mild	Urban
FG05	PWD				66	Male	4	Lewy body	Mild/moderate	Urban
FG06	Carer	59	Female	Spouse	66	Male	4	Lewy body	Moderate	Urban
FG07	Carer	76	Female	Spouse	80	Male	4	Mixed	Moderate	Urban
FG08	Carer	62	Female	Daughter	84	Male	NA	Alzheimer’s	Mild	Urban
FG09	PWD				84	Male	NA	Alzheimer’s	Mild/moderate	Urban
FG10	Carer	72	Female	Spouse	NA	Male	NA	NA	Moderate/severe	Urban

Prior to participation, study participants were guided through the informed consent process, reviewed the participant information sheets and signed a consent form.

Interviewees were prompted to think about whether services were available and satisfactory in meeting their needs and expectations, and why. Focus group participants discussed the emergent themes from the interviews and were invited to express whether these also represented their experiences in order to validate and add breadth to the interview data.

Interviews and focus groups were audio-recorded, professionally transcribed, anonymised and analysed using NVivo 12 [34]. First, three team members (CDP, JO, MA) read the transcripts to familiarise themselves with the dataset. Second, they carried out a thematic analysis of four interviews (two transcripts each coded by two researchers) using a codebook derived from published frameworks of needs of people with dementia and family carers [1, 8, 35, 36] to establish the needs expressed in our sample. Through this process the codebook was iteratively refined to capture additional emergent themes, provide an equivalent degree of granularity across themes and ensure their person-centredness. These discussions raised the analysts’ awareness of their individual perspectives, including the influences of their professional backgrounds, and helped sensitise them to issues that would otherwise not have been considered. The analytic and methodological outcomes of such discussions were documented in notes that complemented the NVivo coding files. Third, in order to safeguard against either dominating the analysis, two members of the team (CDP and JO) analysed the full set of interviews using the final codebook of needs. In those instances when it was felt that the data were ambivalent, the researchers re-analysed them together. In doing so, they also used notes collected around the time of the fieldwork and resolved the tensions through discussion.

Following this, the same two members of the team turned to the conceptual framework to analyse the data related to the need most frequently reported by people living with dementia or their family carers reflecting on the need of the person they cared for. The framework was developed at this stage with two aims: first, to help place variations in care needs along the care delivery process; secondly, to cluster around the theoretical dimensions suggested by candidacy theory and discrepancy theory the different factors in action at each point of the care delivery process that could explain such variations. In the original theories such factors are described in broad and generic terms. The two analysts extensively discussed how to operationalise them, given the specific context in which they were working. Based on these discussions they agreed and then implemented a codebook which used themes stemming from the conceptual framework. It is this final step, the application of the conceptual framework to the most frequently reported need, which is reported here.

## Results

The need most frequently reported for people with dementia, i.e. the need for assistance with going out and about, was used as exemplar to operationalise the conceptual framework. Interestingly, the need identified was neither health nor care related and yet the study participants repeatedly mentioned its importance, as epitomized by a carer: *“It’s not an extra to get out, it’s an essential to get out”* [FG01].

### The need for assistance with going out and about

Dementia may impair memory and spatial and temporal orientation, affecting people’s ability to drive safely, recognise places or follow directions, as expressed by this couple:

*Husband (carer): She gets lost. And easily, and over the last few months, it’s got even worse and she just can’t get on a bus and safely get to the destination. When she’s there she can’t, she gets so easily lost […]So, she doesn’t have the mental map in her head how [town] is laid out.*

*Wife (with dementia): Yeah, but the same when I go the second time. I think there’s something like, where am I? How do you get back? You know, […] go in a shopping centre and I can’t remember which door we had to go back out […]*. *So I don’t go on my own no more. It’s a lot safer not going on my own. [CDP08].*

In light of such difficulties, the need of people living with dementia most frequently reported was for support to go out and about safely, to attend medical appointments, activity groups, or carry out activities of daily living, and, fundamentally, to retain active, meaningful engagement in the community. This need was frequently met by family and friends, often with resignation, which was voiced by a carer:

*Wife (carer): There’s a new dementia café just started up and I got the flyer for that this week […]in [town] which is about three miles down the road.*

*Interviewer: And who is going to arrange transport from home to there?*

*Wife (carer): I would, I would take him to that I think but this is the problem that everything we do I have to take him. [CDP05].*

However, family carers could not always meet the need because of their own health problems, competing care responsibilities, work commitments or simply could not drive. Additionally, not all people with dementia had nearby family or friends to draw upon. In these cases, the person with dementia, and sometimes their carer, needed to seek support. When this need was met, it could lead to high levels of satisfaction as the person regained a sense of connection with the outside world and a resurgence of self-esteem, as articulated by this couple reflecting on the positive impact of the support they were receiving from a local charity:

*Wife (with dementia): My life’s changed so much. The quality of life. […]within the last year and a half, I’ve gone from someone sat here vegetating to someone that’s got a little bit of a life.*

*Husband (carer): Yeah, someone said you’ve gone from what it was, to like a social butterfly, which is quite nice, isn’t it? [CDP08].*

When the need was not met it could leave a person with very limited opportunities for leaving the house safely and lead to social isolation, as for this couple:

*Husband (carer): Well, somebody come out and talked to us about dementia and what services were available and what, you know... there’s a place down in [town] where we can go and sit with other people suffering from dementia and have tea and coffee. But, again, they’ve missed the fact that I’m not mobile and therefore Pauline isn’t.*

*Interviewer: Yes. So the person who came out was telling you places you could go but you haven’t a way of getting there?*

*Wife (with dementia): Yeah. [JO05].*

### Identification of a need

According to candidacy theory, the first step in satisfying a need is the recognition that the need exists. The need for assistance to go out was repeatedly mentioned by the study participants, especially by those living in more rural areas:

*Focus group participant (with dementia): Well for us […] in [rural village] there’s two buses and if you don’t get the one coming back in the afternoon at 12:50 pm, there’s nothing else. And it’s very […] And if you don’t drive or have access to somebody who drives then it’s a lot more difficult. [FG02].*

By contrast, other needs (e.g. help with personal care) seemed to be less readily recognised by those living with dementia or their carers. For example, the eventual trigger for one husband to seek help from formal services had only occurred after years of caring for his wife, when he realised he needed more support. Even then, it was his daughters, not himself, who arranged this.

*Interviewer: So three or four months ago, when you thought you could do with calling on official sources of help, where did you turn to get that advice on finances, or services?*

*Husband (carer): Well, I didn’t, actually. It was my daughters. [JO01].*

Some carers did not identify their ‘need’ and expressed a stoical attitude (*“I do what I have to do and just get on with things”* [CDP14]). Spouses seemed more likely to want to keep caring by themselves than adult-children, due to a wish to protect the person with dementia from poor care and stigma, to adhere to social norms, or simply because they felt it was the right thing to do.

### Availability of a service

The following phase in the care delivery process was aimed at identifying a service(s) that could help with going out. The services sought within our sample included public transport as replacement for driving, minibus services for the ‘disabled’, ambulance transport and personal support to accompany someone.

In some cases, an appropriate service did not exist (or was not located), as in this example from a daughter-in-law, whose mother-in-law had moved to live close by, since she had become isolated in the community where she had lived for many years:

*Daughter-in-law (carer): I tried to get her to go to a group down there with the Alzheimer’s Society which, when I went with her, was fine. When she tried to go by herself, she couldn’t find her way there and it was somewhere that she did know the area but she couldn’t find her way there. She couldn’t find her way back, which caused a problem, so she wasn’t able to go. I had nobody to take her so that meant she couldn’t go. […] And I think that’s what’s made the difference up here, is I can take her, I can pick her up and she stays there. [CDP09].*

The experience of a visually impaired husband caring for his wife, diagnosed with dementia, shows the difficulties that users may experience in identifying a service. They had only very recently discovered a transport service which would take them where they needed.

*Husband (carer): There is a free, well not a free service but it’s for hospital appointments. Now I rang them yesterday, Monday, to ask if I could use that service to go and visit me brother in the care home and they said yes, we can allow that. So I’ve also joined an organisation called [organisation] in [town] and we can use it to go to their dos. So we’re starting to broad... we were there last...*

*Wife (with dementia): Last week. [JO05].*

In cases where a service did exist, issues of access were raised.

### Access to the service

Interviewees’ accounts exposed a range of barriers to accessing transport services. We conceptualise their experiences using the conditions suggested by candidacy theory.

#### Offer and resistance

In some instances, the service offered was ‘resisted’ by prospective service users who considered it unacceptable, as suggested by a carer:

*Daughter-in-law (carer): There’s a taxi service taking them all home now [from local venue hosting activities for people with dementia] as well. I think that’s the big problem for a lot of people as well, elderly people, is transport because it’s okay saying they can get taxis and they’ve got their attendance allowance, it’s there to pay for taxis. Elderly people don’t like to spend money on things like that and they won’t spend money on it. And it’s a big issue. If there was a bus that went round and picked them all up … [CDP09].*

The taxi service was presumably intended to facilitate people with dementia accessing the activity groups. However, according to this interviewee, this was not a culturally acceptable offer to people from a generation who were used to being frugal. The expected users were reluctant to take up the offer and, in fact, did not use the service, leaving their need to go out and access social contact unmet.

#### Navigation

Costs affected the ease of navigation of services, as indicated by an interviewee who was also a support worker for a local organisation:

*Focus group participant (carer): So we take people with dementia out, to try to keep them into the community. We take them shopping or cinema or any, anything they want. […] It’s £17.50 an hour and you’ve got to have 2 hours so it’s very expensive and the Social Services don’t like paying for that at all. [FG04].*

Additionally, for some participants, the effort required to access services proved a serious hurdle. One couple both experienced memory and other health problems, so hospital transport represented their only option for traveling to their medical appointments, yet they struggled to arrange this:

*Husband (carer): And what we’ve been finding problems with is, we use hospital transport because we can’t get up ourselves, and a while ago, because of all the cutbacks, was that they weren’t either allow[ing] me to go with Sheila, or Sheila to come along with me to my hospital appointments [...]. but, Professor X said, ‘Sheila simply isn’t allowed to come up [on her own], she won’t be able to say what it is.’ So now I have to say at the beginning to the hospital transport, that it’s dementia but it’s all hit and miss, if you see what I mean.*

*Wife (with dementia): They do allow the dementia patients, don’t they, to go in the ambulance to go and see a Consultant on their own?[…] You have got to force, to get to the appointments.*

*Husband (carer): So you’ve got to be quite, you’ve got to be really, really assertive and say to them, I need to go, and they will think of dozens of reasons why you can’t go. So it’s not an easy service to, to use.*

*Wife (with dementia): Because I can’t go on [my own]. [CDP08].*

This example shows not only the effort required to negotiate use of a crucial service but also how much determination and confidence is needed to keep appointments and how understandable it would be if appointments were missed, in such circumstances.

#### Adjudication

The continuation of the quote above provides an example of ‘adjudication’, i.e. the process through which the professional makes a judgement about a candidacy claim. Based on the experience of this couple, it does not seem possible to understand the criteria used by the provider in deciding whether to give them access to the transport service:

*Husband (carer): And I, every time I phone up, I think, oh, here we go again. You know, it’s not going to be a matter of just booking it up and I’m worried about Sheila going up, I’m worried about the lumbar puncture, now I’m having to worry about, can I go with her? And sometimes they say, ‘Yes, it’s no problem.’ Another person you get hold of and it’s, ‘Well I don’t know.’*

*Wife (with dementia): I’m not going then. I said, ‘I won’t go unless I’ve got somebody with me’ because I can’t, I can’t go on my own, because I know that when I get there and they ask me all these questions, I can’t, I can’t, and I get muddled and I thought, no, no, I’m not going that way. So I said, ‘I won’t go then’. [CDP08].*

At times, the couple’s attempts to arrange transport were hindered, whereas at other times their request was met without opposition. This inconsistent behaviour caused stress and distress for the couple and affected their attitude to attending their appointments.

A more generous adjudication process was experienced by the visually impaired man caring for his wife [JO05]. The couple managed to make arrangements with the hospital transport service to visit a family member in a care home and to go to social events, so enabling them to be socially active. We do not know why the service accommodated their request. It may have been partially influenced by the ability of the husband to fluently explain their needs (see ‘appearance’) as he was a former welfare worker who had made cases for others to access services during his career.

#### Appearance

Appearance also proved to influence service access. Those living with dementia experience cognitive problems which may impair their ability to articulate the issue for which help is sought. Service providers may not appreciate the difference dementia makes to their abilities and then consider a request for service unjustified, as exemplified by this account of a woman with dementia who tried to arrange for her local pharmacy to deliver the medication needed by her husband.

*Wife (with dementia): I went to the chemist and because I’d changed chemists to the local one just down the road. And I said, ‘Danny’s had a stroke and, and, and spinal injury, and I need the tablets delivered, because I can’t come down, because there’s a big hill to go down, and by the time I was going down the hill and come back up, my breathing, because I’ve got lung problems as well, I couldn’t. I went down the chemist and I tried to explain to him, and he said, ‘You’ve got to ring NHS.’ And I said, ‘But how, how do I? I need these tablets delivered. I can’t take them.’ And he said, ‘Well, you’ll have to sort it out yourself.’ And then that was a chemist! I, I broke down in tears in the chemist, they put me in this little room and he told me that he couldn’t help me, I have to ring myself. [...] He said, ‘It’s a free organisation and you’ll have to go and get help.’ And I got home, and I thought, ‘Well what do I do now?’ So, I didn’t. I never took, collected the tablets for a week and we just went without, because I just couldn’t get down there, you know, it was a real struggle that chemists don’t realise. [CDP08].*

In this instance, the woman, who had dementia as well other health problems, was unable to collect and carry the medication. She was left to arrange its delivery by phoning the home delivery service. However, due to her cognitive impairment, she felt that she was not able to do this. Her account provides evidence of the complexity of ‘appearing’ at a service and articulating needs by somebody living with dementia. When such complexity cannot be navigated, appearance does not happen, as in this instance.

#### Permeability

The same quote also illustrates how permeability affects access. In the case reported, the service seemed too rigidly organised to accommodate the requests of somebody with complex needs (“*He said, ‘It’s a free organisation and you’ll have to go and get help.’”)* and the service permeability seems to be low. In other instances, the restricted schedule of public transport services also provided an example of lack of permeability, as the service was not available when needed and, hence, did not help people to go out:

*Interviewer: Have you been able to get to anything that they’ve told you about or you’ve stopped because you can’t get there?*

*Wife (with dementia): We have to rely on the local bus service. That finishes at 6 o’clock, for us.[JO05].*

A permeable service, i.e. accessible when needed, could make a substantial difference to people’s well-being. In the example below, a woman recounts how the ‘PA’ service she accessed using her personal budget, was highly flexible and had enabled her to get out shopping for the first time in many months:

*Wife (with dementia): The hours they [local provider] gave us is brilliant, because they come, they take us out, they take me shopping, they help me buy clothes, you know, and, and I couldn’t have had … before that, I would say six months, six, seven months, I weren’t even going out the house. [CDP08].*

#### System-level influences

According to candidacy theory, availability and suitability of resources shape service access. A couple recounted how austerity had affected basic service provision and affected their candidacy claims:

*Husband (carer): We use hospital transport because we can’t get up ourselves, and because of all the cutbacks, a few months ago they were like really reluctant to allow me either to go with Sheila, or Sheila to come along with me to hospital appointments. [CDP08].*

This squeeze on a formal service can be contrasted with the access given to the visually impaired carer trying to visit his brother in the care home [JO05]. While the ambulance service needed by the couple in CDP08 was a stretched resource, the hospital car service, staffed by volunteer drivers, negotiated by the interviewee in JO05 had more generous resources: the first one seems to reflect the rigidity of a bureaucratic organisation operating with scarce resources, such as the NHS, the latter may have existed in a more flexible system of the type associated with volunteers.

### Use and satisfaction with the service

Some participants described succeeding in making arrangements to meet their need to go out, i.e. they moved from the step of negotiating access to using the service. This led to varying degrees of satisfaction, dependent on the experience of a specific service in comparison to the user’s expectations. For example, an interviewee articulated why hospital transport was deemed poorly organised and, hence, unsatisfactory. He was not having to use it at the present time as, although he could no longer drive, his wife was still able to. However, it sounded as if he was already thinking about its unsatisfactory nature and feeling sorry for those who had to rely on it.

*Husband (with dementia): Luckily my wife still drives, so we have mobility. Now some people […] they’re having to either get on a bus or sort it out for themselves or get a taxi. It’s either funding it or if they haven’t got the, the money, or getting an ambulance to go around the bloody [name of the area] to get you to wherever your appointment is. So it’s not just a couple of hours job, it’s nearly a day’s job. [CDP13].*

In his account, there is a discrepancy between his expectations around efficient use of time and the reality of having to rely on ambulance transport that takes hours to collect a series of patients on its way to the hospital.

However, most users were satisfied with their transport arrangements as long as the service was available, accessible and would allow them to carry on with their personal and social life. In the following extract, the husband reflected on the additional opportunities from which – he hopes – they will benefit, having finally found a means of transport to access local activities:

*Husband (carer): And now I’m looking outward, as I say, we’ve joined [organisation] in [town] and I know some of them so, and it is good. I want to get Pauline out the house as much as often. [JO05].*

Users’ satisfaction was high when services were person-centred and flexible enough to provide the support that those living with dementia required on the day, rather than being bound to a rigid set of tasks.

*Partner (carer): Now this company, if you don’t want them to clean or something, they’ll give him a run along to Sainsbury’s to buy your present or something, or Marks’ or something like that. So, it’s little things that are very important, as well as the practicalities of dressing somebody and, you know. So I feel very, I feel much more, I think we both do, secure in that respect. [CDP04].*

## Discussion

Extant literature acknowledges that the needs of people with dementia are wide-ranging and often remain unmet [7–13, 37], despite attempts to improve care. We propose a framework analytically structured in five phases mirroring the care delivery process which combines candidacy and discrepancy theories to explain variation in whether, when and why needs of people with dementia are met. The framework pays attention to the gap between service availability and access (in accord with candidacy theory) and between service use and user satisfaction (in accord with discrepancy theory).

In this work, the identification of candidacy was considered a prerequisite for approaching the care delivery system and, in doing so, differs from the traditional candidacy framework. If users do not (or are not supported to) acknowledge their need in the first place, they would not look for support or try to use services. Thus, a need would remain unmet regardless of local service provision. The process of identification by those living with dementia of their need to go out safely was straightforward. Consistently with previous research [38, 39], interviewees were able to anticipate or recall the consequences of not being able to satisfy this need, for example in terms of social isolation. However, recognition of other needs may be more complex and influenced by social norms, stigma, denial and individual readiness to use services [17–20].

The second phase of the framework aimed at establishing whether formal services or informal support was available to meet the need. Service provision to go out seemed limited, echoing the results of previous studies showing lack of adequate service provision in rural areas and for people in the early stages of dementia [18, 19, 40, 41].

The third phase investigated access through the lenses of candidacy theory, which proved useful to unpack the individual-, service- and system-level factors enabling or preventing access to transport services for people with dementia. The candidacy framework has previously been adopted to explore the challenges of navigating healthcare services in order to secure a diagnosis of dementia [42]. Here, by embracing a need-led approach, candidacy was operationalised with respect to satisfying a specific need rather than accessing a particular service. In doing so, the framework was applied across services spanning different sectors and illustrated the complex constellation of services required to address this single need.

At the individual level, personal preferences and values, cognitive and physical skills and financial resources were the key factors that shaped and determined navigation, appearance, and offer and resistance when people with dementia and family carers tried accessing transport services.

Ease of navigation was influenced by the cognitive skills required to identify, process and use information to locate services. The interviewees recalled that access to transport services or to home delivery of medication required a self-referral to the provider, usually through a phone call. However, knowing who to contact and how to contact them could demand considerable cognitive effort, constituting a hurdle for many people with dementia. Equally, navigation could be problematic for carers, many of whom were also contending with their own health problems or disabilities. The health and care system appeared to assume cognitively and physically competent and agile service users, rather than taking into account that people needing assistance to get out have inevitably some degree of disability that would demand accessible, user-friendly information and communication systems. Despite the present analysis investigating a demographically stable and homogeneous population, these results are aligned with evidence from previous applications of candidacy theory in the context of disadvantaged populations [21], ethnic minorities [42] and migrant populations [43]**,** highlighting how issues associated with the ‘information work’ (identifying, filtering, retaining and acting upon relevant information) [44] cross population sub-groups.

As expected from previous applications of the candidacy framework, financial resources determined affordability of services, shaping navigation [21, 29, 31].

Cognitive and physical skills affected whether people with dementia were able to articulate clearly and credibly their candidacy claim and their ‘appearing’ at services. Hence, this expands the possible determinants of appearance, beyond users’ socio-economic characteristics [21], their conceptualisation of illness identity [28] and their awareness or perception of organisational constraints within which care is provided (e.g. time restriction within consultations) [45].

The category of offer and resistance was shaped by individual preferences or values, influenced by cultural context, with some services being turned down even in cases where the need was identified, as exemplified by the refusal to use a taxi service to attend local groups due to generational attitudes. This resonates with previous work highlighting that resistance is the individual’s response to care which is not truly patient-centred and does not take into account the compounding intersections of users’ identities (e.g. gender, cultural ideals [31, 42]).

Flexibility of services and referral or decision criteria used by service providers when assessing a candidacy claim represented the service-level factors contributing to, respectively, permeability and adjudications. In the example of hospital transport, lack of resources affected permeability: as transport is a means to an end (e.g. to keep appointments), service users do not have flexibility to accommodate their candidacy to fit restricted schedules. Our work shows that in addition to referrals [28] and appointment and booking systems [31, 45], service flexibility also represents an important feature of permeability.

Participants’ accounts highlighted how providers’ adjudication processes spanned from being inconsistent and based on ambiguous eligibility criteria, as in the case of hospital transport, to being rigid and overly complicated, as in the case of home delivery of medications, and unquestionably represented local operating conditions (e.g. resource constraints) within which services operated. The outcomes of such adjudications carried out by administrative staff dealing with service requests seemed to be determined a priori, regardless of the legitimacy or deservingness of the users’ claim (a more subjective criterion which was used by Professor X, in CDP08).

Contrary to previous work which has collapsed local operating conditions into generic contextual local factors [28, 46], this work specified three types of operating conditions affecting access to transport services for people with dementia. Firstly, budget cuts determined whether instances of candidacy could be met. Secondly, local organisational (and commissioning) frameworks were not designed to support users articulating their candidacy and rather worked against it, for example by not providing users with information on eligibility criteria. Lastly, the dominant disease-led, siloed culture within which the care providers operate seemed to clash against how users framed their candidacies, a need-focused process and not diagnosis- or service-led. The local care delivery system seemed, in practice, to be poorly equipped to assess candidacy claims in a person-centred way.

The fourth phase investigated the actual experience of service use and led to the final phase focusing on whether and why the experience of using the service was satisfactory. Discrepancy theory was operationalised using Donabedian’s determinants of care satisfaction. Outcomes indicated overall satisfaction with care, and structure and process provided insight into the nature and location of the deficiencies or strengths at the service-level to which the outcome might be attributed [33]. With respect to the needs of people with dementia to get out, dissatisfaction was associated with services that, from the users’ perspective, were inconveniently scheduled (structure) or too rigidly organised (process).

The work presented in this article is relevant for three different audiences. Firstly, the conceptual framework is in itself a heuristic tool which could be of interest for any professional or policy maker with responsibilities spanning across the levels and factors that enable or hinder satisfaction of care needs. Individual-level factors can be addressed by health and care practitioners through the application of genuinely person-centred care which should acknowledge and be shaped by the variety of preferences, skills and circumstances that affect whether and how patients and carers frame, and re-frame, their need(s) and their candidacy instances. Service-level factors can be tackled by providers, e.g. by addressing the lack of coordination or integration of services or by improving the transparency of eligibility criteria. System-level factors are influenced by high-level, strategic choices made by decision makers across and within sectors, for example in terms of budget allocations.

Secondly, the application of the framework discussed in this article has specific implications for professionals and policy makers with an interest in dementia care. Person-centred provision of care for those living with dementia, a long-term progressive condition characterised by a complex and evolving set of needs (biological, emotional, cognitive, social and practical), requires great flexibility in the offerings of formal services alongside financial mechanisms (e.g. in the form of direct payments) to support choice and control by service users over which services to access at a specific point in time, if needs are to be effectively met.

Lastly, the research community is a further potential beneficiary of this work. By providing a configuration of factors that can explain the satisfaction of care needs, the conceptual framework represents a theory. As such, it can be used in the improvement science field, for example to inform the design of interventions, or it can be tested out as a middle-range theory in realist evaluations, or as a programme theory in theory-driven evaluations more generally, to explain how and why interventions are effective.

We recognise some methodological limitations of this work. The direct experiences of people living with dementia are underrepresented because of difficulties in participant recruitment. The majority of interviews with people with dementia took place with a relative present, which may have had an impact on the interview dynamic and the freedom with which the persons with dementia shared their feelings or opinion.

## Conclusions

A framework combining candidacy and discrepancy theories, mapped onto the care delivery process, can help explain variations in satisfaction of care needs. Firstly, it acknowledges the importance of need identification as a prerequisite to need satisfaction. Secondly, it surfaces the laborious process of (re)framing claims of candidacy experienced by care users and identifies causes of variations in satisfaction of needs arising around the point of service access. Then, it conceptualises care satisfaction in terms of discrepancies between individual expectations around a service and the actual experience of using that service, assessed in terms of service structure, process and outcomes. In doing so, the framework offers a comprehensive heuristic tool to identify and understand enablers and barriers to the provision of personalised, need-focused care and can be applied to complex, long term conditions.

## Supplementary information

**Additional file 1.** Guide for focus group with people with dementia and family carers.

**Additional file 2.** Guide for interview with family carers.

**Additional file 3.** Guide for interview with people living with dementia.

## Data Availability

The datasets used and/or analysed during the current study are available from the corresponding author on reasonable request.
