# Supplementary material for: A need-based, multi-level, cross-sectoral framework to explain variations in satisfaction of care needs among people living with dementia
Source: BMC Health Serv Res. 2020 Jul 15;20:657. doi: 10.1186/s12913-020-05416-x (PMC7364635; doi:10.1186/s12913-020-05416-x)
Supplement: Supplementary file 1 — Additional file 1. Guide for focus group with people with dementia and family carers. [file 12913_2020_5416_MOESM1_ESM.docx]

**GUIDE FOR FOCUS GROUP WITH PEOPLE WITH DEMENTIA AND FAMILY CARERS**

|  |  | **Prompts for the discussion** |
| --- | --- | --- |
| **Preamble** | Over the last few months the project team has been talking with people with dementia and their families in [name of the area]. Now we would like to share what they have told us and ask if this is similar to your experiences … |  |
| **Needs & service use** | Share extracts from interviews, providing context and some background information for each | With reference to each quote or homogeneous groups of quotes   - Does this ring true? - Did/do you have similar needs? Have you used similar services? What was your experience? What worked well for you? What did not work so well? - Was there anything that you felt you needed but no service was providing? - Thinking of your experience, is there any particular service or aspect of a service that should be improved as a matter of urgency? Why? |
| **Conclusion & thank you** | Ask if participants would like to add anything else |  |
