# Supplementary material for: A need-based, multi-level, cross-sectoral framework to explain variations in satisfaction of care needs among people living with dementia
Source: BMC Health Serv Res. 2020 Jul 15;20:657. doi: 10.1186/s12913-020-05416-x (PMC7364635; doi:10.1186/s12913-020-05416-x)
Supplement: Supplementary file 2 — Additional file 2. Guide for interview with family carers. [file 12913_2020_5416_MOESM2_ESM.docx]

**GUIDE FOR Interview WITH Family carerS**

|  | **Questions** | **Prompts** |
| --- | --- | --- |
| **Preamble** | I’d like to ask you to tell me more about the experience of dementia care that [name of person with dementia] and you are receiving… |  |
| **When everything started** | 1. Can you tell me about the time when you first started to worry that something wasn’t right? |  |
| **Trigger(s) to approach services** | 1. So, what did you do? |  |
|  | 1. And then, what happened next? | - Did you go and see your GP? What did the GP suggest to do? How did you feel about that? |
| **Diagnosis** | 1. When was s/he diagnosed? | - Who made the diagnosis? Did s/he get any test done? How did you feel about this? |
| **After the diagnosis** | 1. What happened after they told you that s/he has dementia? | - Did someone give you information about dementia? - Were you offered access to some services? Were they helpful? Were you happy with the services you were offered at the time? Was the help offered at the right time for you? Offered in the best place? - Did you feel you needed more help? Different kind of help? - Were you offered a carer’s assessment^^[[1]](#footnote-1)^^? Did anything happen as a result of the carer’s assessment? - Are you on the carers’ emergency support register? - Were you offered contact with a dementia advisor^^[[2]](#footnote-2)^^? Do you know this service? If you know the service, who referred you on to them? Were they helpful? |
| **Progression of the disease** | 1. Since the diagnosis, what sort of things have changed? | - Has dementia got worse? - Have your personal / family circumstances changed? - Did you feel over time you needed more help? Of what sort? Or different type of help? Did you find the help you needed? Where? Thanks to whom? |
| **Urgent, out of hour care** | 1. Have you ever needed urgent help at night or over a weekend? If so, what happened? Where did you turn for help and what help did you get? 2. If you needed urgent care at night or over a weekend, what would you do? | - Thinking of [name of person with dementia], did you need urgent care for a physical problem (e.g. UTI? a fall?)? or for something related with dementia, e.g. challenging behaviour? - In case you needed help for a not life-threatening problem not requiring an A&E, what would you do? - Have you ever needed emergency replacement care? |
| **Now** | 1. What services are you as a carer now using? | - Are you happy with these services? Is there anything that you would like to change about them? What do you like most/least about your local services? |
|  | 1. What services is [name of person with dementia] now using? | - Are you happy with these services? Is there anything that you would like to change about them? What do you like most/least about your local services? |
| **Concluding comments** | 1. What are your views on the needs of a person with dementia? 2. What are your views on the needs of someone who is caring for a person with dementia? | - Throughout your journey, can you think of a moment/situation when you felt you needed help and you could not find it? |
| **End** | 1. Is there anything else you would like to add? |  |

1. A carer’s assessment is done by the local Council and looks at how caring affects your life, including for example, physical, mental and emotional needs, and whether you are able or willing to carry on caring. [↑](#footnote-ref-1)
2. A Dementia Advisor is a social worker from the Alzheimer's Society and they help carers and people with dementia to access local services. [↑](#footnote-ref-2)
