# Supplementary material for: A need-based, multi-level, cross-sectoral framework to explain variations in satisfaction of care needs among people living with dementia
Source: BMC Health Serv Res. 2020 Jul 15;20:657. doi: 10.1186/s12913-020-05416-x (PMC7364635; doi:10.1186/s12913-020-05416-x)
Supplement: Supplementary file 3 — Additional file 3. Guide for interview with people living with dementia. [file 12913_2020_5416_MOESM3_ESM.docx]

**GUIDE FOR Interview WITH PEOPLE LIVING with dementia**

|  | **Questions** | **Prompts** |
| --- | --- | --- |
| **Preamble** | I’d like to ask you to tell me more about your experience of dementia care… |  |
| **When everything started** | 1. Can you tell me about the time when you first started to worry that something wasn’t right? |  |
| **Trigger(s) to approach services** | 1. So, what did you do? |  |
|  | 1. And then, what happened next? | - Did you go and see your GP? What did the GP suggest to do? - How did you feel about that? |
| **Diagnosis** | 1. Do you remember when you got your diagnosis? Do you remember having the assessment e.g. completing memory tests or having a scan? Who gave you the diagnosis? | - How did you feel about the tests? - How did you feel about being given the diagnosis? |
| **After the diagnosis** | 1. What happened after you were told that you have dementia? | - Did someone give you information about dementia? - Were you offered access to some services? What services? Were they helpful? Were you happy with the services you were offered at the time? Was the help offered at the right time for you? Offered in the best place? - Did you feel you needed more help? Different kind of help? - Were you offered contact with a dementia advisor^^[[1]](#footnote-1)^^? Do you know this service? If you know the service, who referred you on to them? Were they helpful? |
| **Progression of the disease** | 1. Since the diagnosis, what sort of things have changed? | - Has your memory or other problems with thinking got worse? - Have your personal / family circumstances changed? - Has the dementia affected your independence or your social life? - Did you feel over time you needed more help? Of what sort? Or different type of help? Did you find the help you needed? Where? Thanks to whom? |
| **Urgent, out of hour care** | 1. Have you ever needed urgent help at night or over a weekend? If so, what happened, where did you turn for help and what help did you get? 2. If you needed urgent care at night or over a weekend, what would you do? | - What sort of thing was it that required urgent care? (an infection or a fall?) or a blackout or collapse? - If you needed help for a non life-threatening problem not requiring an A&E, what would you do? |
| **Now** | 1. What services are you using now? | - Are you happy with these services? Is there anything that you would like to change about them? What do you like most/least about your local services? |
| **Concluding comments** | 1. What are your views on the needs of a person with dementia? | - Throughout your journey, can you think of a moment/situation when you felt you needed help and you could not find it? |
| **End** | 1. Is there anything else you would like to add? |  |

1. A Dementia Advisor is a social worker from the Alzheimer's Society and they help carers and people with dementia to access local services. [↑](#footnote-ref-1)
